# Conventional Manufacturing by Pouring Versus Additive Manufacturing Technology of β-Tricalcium Phosphate Bone Substitute Implants

**DOI:** 10.3390/biomedicines12081800

**Published:** 2024-08-08

**Authors:** Tanja Zöller, Hagen Schmal, Matthias Ahlhelm, Hermann O. Mayr, Michael Seidenstuecker

**Affiliations:** 1 G.E.R.N. Tissue Replacement, Regeneration & Neogenesis, Department of Orthopedics and Trauma Surgery, Medical Center-Albert-Ludwigs-University of Freiburg, Faculty of Medicine, Albert-Ludwigs-University of Freiburg, Hugstetter Straße 55, 79106 Freiburg, Germany; zoellertanja@gmail.com; 2 Department of Orthopedics and Trauma Surgery, Medical Center-Albert-Ludwigs-University of Freiburg, Faculty of Medicine, Albert-Ludwigs-University of Freiburg, Hugstetter Straße 55, 79106 Freiburg, Germany; hagen.schmal@uniklinik-freiburg.de (H.S.); hermann.mayr@uniklinik-freiburg.de (H.O.M.); 3 Department of Orthopedic Surgery and Traumatology, Odense University Hospital, 5000 Odense, Denmark; 4 Fraunhofer Institute for Ceramic Technologies and Systems, IKTS, Maria-Reiche-Str. 2, 01109 Dresden, Germany; matthias.ahlhelm@ikts.fraunhofer.de

**Keywords:** sintering, β-TCP, additive manufacturing, freeze foam, hybrid bone, biocompatibility, bone replacement

## Abstract

The aim of the study was to compare conventional sintering with additive manufacturing techniques for β-TCP bioceramics, focusing on mechanical properties and biocompatibility. A “critical” bone defect requires surgical intervention beyond simple stabilization. Autologous bone grafting is the gold standard treatment for such defects, but it has its limitations. Alloplastic bone grafting with synthetic materials is becoming increasingly popular. The use of bone graft substitutes has increased significantly, and current research has focused on optimizing these substitutes, whereas this study compares two existing manufacturing techniques and the resulting β-TCP implants. The 3D printed β-TCP hybrid structure implant was fabricated from two components, a column structure and a freeze foam, which were sintered together. The conventionally fabricated ceramics were fabricated by casting. Both scaffolds were characterized for porosity, mechanical properties, and biocompatibility. The hybrid structure had an overall porosity of 74.4 ± 0.5%. The microporous β-TCP implants had a porosity of 43.5 ± 2.4%, while the macroporous β-TCP implants had a porosity of 61.81%. Mechanical testing revealed that the hybrid structure had a compressive strength of 10.4 ± 6 MPa, which was significantly lower than the microporous β-TCP implants with 32.9 ± 8.7 MPa. Biocompatibility evaluations showed a steady increase in cell proliferation over time for all the β-TCP implants, with minimal cytotoxicity. This study provides a valuable insight into the potential of additive manufacturing for β-TCP bioceramics in the treatment of bone defects.

## 1. Introduction

There is no consensus on the definition of a “critical” bone defect; it is generally considered to be a defect of such magnitude that it cannot heal spontaneously; therefore, it requires surgical intervention beyond stabilization of the bone by osteosynthesis, such as autologous bone grafting [1]. The underlying causes of relevant bone defects are diverse. In most cases, the defect occurs as a result of debridement after blunt force trauma when non-vital bone fragments are removed. Sharp force can also lead directly to bone loss [2]. The etiology of bone defects also includes excision of bone tumors and debridement after bone infections. Although bone tumors themselves have their highest incidence in the 10–14 age group at 10.6% [3], bone is the third most common site for metastases from primary solid tumors, including bronchial, colorectal, prostate, and breast cancer [4], which can cause pathologic fractures and necessitate the treatment of bone defects [5]. The current gold standard for treating bone defects is still autologous bone grafting [6]. This involves harvesting the patient’s own bone from another site, often the iliac crest, and transplanting it to the defect site.

In 2021, 43,118 bone grafts were harvested in Germany [7]. However, this treatment method results in a donor-side defect, which is associated with significant postoperative morbidity and can cause chronic pain at the donor site [8,9]. The amount of autologous bone available for transplantation is very limited. The second surgical field is also associated with an increased risk of postoperative wound infection [9]. In addition, bone graft harvesting results in prolonged operative time and thus increases the risks associated with general anesthesia for patients, primarily cardiovascular, respiratory, and renal complications [10].

The cost of treating bone defects also increases due to the longer duration of surgery; taking into account anesthesia, including medication and personnel, as well as the surgical team and materials, the additional cost per operation amounts to hundreds of euros [11]. These disadvantages can be avoided with alloplastic bone replacement, which involves the transplantation of synthetic foreign material. The relative use of alloplastic bone graft substitutes in the treatment of bone defects has increased significantly: from 11.8% in 2008 (10,163 cases in total) to 23.9% in 2018 (23,838 cases in total) [12].

The optimization of these bone substitutes is the subject of ongoing research, including studies on the release of growth factors through the material [13,14,15] or the integration of stem cells into the material [13,15,16,17]. Bone graft substitutes are already used in a variety of specialties, including orthopedics, dentistry, and oral and maxillofacial surgery. The use of metals to make plates, screws, and endoprostheses is common in orthopedics, with titanium being the most commonly used material, followed by magnesium and strontium [13]. In endodontics, bioceramics are used for various procedures, such as root canal sealing [18]. In orthopedics, bioceramics are used to treat traumatic defects, such as fractures of the tibia, acetabulum, or distal radius [19,20,21], and to fill defects after resection of benign bone tumors [19,22,23]. These bioceramics are mostly based on calcium phosphates such as hydroxyapatite or beta-tricalcium phosphate (β-TCP). This paper focuses on the latter. Conventional sintering is compared with current additive manufacturing techniques, and the results are evaluated in terms of mechanical strength and biocompatibility.

## 2. Materials and Methods

### 2.1. Materials

Pure ethanol was purchased from VWR International (VWR International GmbH, Darmstadt, Germany). Dulbecco’s Modified Eagle medium nutrient mixture (DMEM/F12), penicillin/streptomycin, Dulbecco’s phosphate-buffered saline (PBS), trypsin-EDTA 0.5%, and trypan blue staining were purchased from Gibco (Grand Island, NE, USA). Ethidium D-III and calcein-AM were purchased as part of a live/dead cell staining II kit (PromoCell, Heidelberg, Germany). MG-63 (ATCC-CRL 1427) cells were purchased from ATCC. The cell proliferation reagent WST-I was purchased from Roche Diagnostics (Basel, Switzerland). The LDH Assay (Cytotoxicity Detection Kit) was purchased from Sigma, now Merck (Darmstadt, Germany)

### 2.2. Manufacturing Processes Used

#### 2.2.1. Manufacturing via Sintering of the β-TCP Ceramics Used

An established manufacturing method for β-TCP implants, which allows their use as bone anchors in anterior cruciate ligament (ACL) reconstruction and as bone replacement [24,25], consists of the following steps. The industrially produced β-TCP was mixed with a porogen by pouring; it was then sintered, calcined, and cleaned. The green body was prepared by mixing α-TCP and TCP (Merck AG, Zug, Switzerland) in a ratio of 4:1 and adding polyacrylic acid (Art. No. 81132, Fluka, Buchs, Switzerland; MW = 5.1 kDa) as a porogen. It was then sintered at 1250 °C for 4 h at a heating and cooling rate of 1 °C/min. Finally, the green body was calcined at a temperature of 900 °C to remove any organic residues and cleaned with ethanol to remove combustion residues [26]. This manufacturing method was used in the industrial production of β-TCP implants in the above-mentioned procedure; accordingly, it is defined as a conventional manufacturing process in this paper [24]. Due to its use as a press-fit anchorage in the bone, this β-TCP implant is shaped like a cylinder, with a diameter of 7 mm, a total length of 25 mm, and a rounded end (cylindrical part with a length of 21.5 mm). According to the medical classification, it is a microporous β-TCP due to the average pore size of 5 µm, and therefore, this implant is henceforth also referred to as a microporous β-TCP implant.

#### 2.2.2. Additive Manufacturing of the β-TCP Ceramics Used

The 3D printed β-TCP implant consists of two components that are joined together in the final manufacturing step and is therefore referred to as a hybrid structure. Both components are made of hydroxyapatite calcined at 900 °C for 2 h. First, the column structure is fabricated using ceramic additive manufacturing vat photopolymerization (VPP). The calcined hydroxyapatite is mixed with solvent, a mixture of synthetic resins, and a photoinitiator. This suspension can now be solidified layer by layer using a UV laser, with a projection of the contours of the desired scaffold replacing a template [27]. The second component of the hybrid structure is a freeze foam. This is produced by foaming a suspension of the ceramic powder, water, a solvent, polyvinyl alcohol as a binder, and an additive for modified rheological properties and pH in a freeze dryer by lowering the ambient pressure and then freezing it. When the ambient pressure is further reduced, the frozen water sublimates and a solid ceramic foam is formed after the subsequent heat treatment [27]. To implement the hybridization, the freeze foam suspension was placed in cylindrical rubber molds, the columnar structure was inserted, and both were placed together in the freeze dryer for the freeze-foaming process. Finally, the final hybrid structure was sintered in the same manner as the conventionally fabricated β-TCP implant. Sintering was performed at 1026.85 °C (1300 K) for 1.5 h after pre-sintering the columnar structure to compensate for its shrinkage during sintering. In this step, the initial hydroxyapatite is transformed into β-TCP. The structure and the two components of the hybrid structure are modeled on the architecture of human bone, which consists of solid cortical bone as the outer framework and an internal latticework of cancellous bone [27]. An overview of the sample characteristics and dimensions is shown in Table 1.

### 2.3. Characterization of the Resulting β-TCP Scaffolds

#### 2.3.1. Characterization of Porosity

Micro-computed tomography: The acquisition of micro-computed tomography images of the microporous β-TCP implant and the macroporous cylindrical β-TCP implant from curasan (curasan AG, Kleinostheim, Germany) of the 3D printed β-TCP implant allows clear size characterization and mapping of the pore distribution. The “Foam Structure Analysis” software VGStudio Max v3.0 (Volume Graphics GmbH, Heidelberg, Germany) was used to precisely determine the porosity of the hybrid structure based on computed tomography images. The required computed tomography images were acquired with the CT-Compact (Procon X-ray GmbH, Sarstedt, Germany) and µCT 100 (Scanco Medical, Brüttisellen, Switzerland). The parameters for µCT were energy 90 kVp, intensity 44 µA (4 W), voxel size 2 µm, field of view 7.4 mm, and integration time 5000 ms. Mercury porosimetry: Porosimetry, performed with the Pascal 140 and 440 mercury porosimeters (Porotec 140/440, POROTEC GmbH, Hofheim, Germany), allows the porosity of the microporous β-TCP implant to be precisely determined and the pore size to be measured. The measurements were performed as described elsewhere [28].

#### 2.3.2. Mechanical Testing

**Figure 1:**
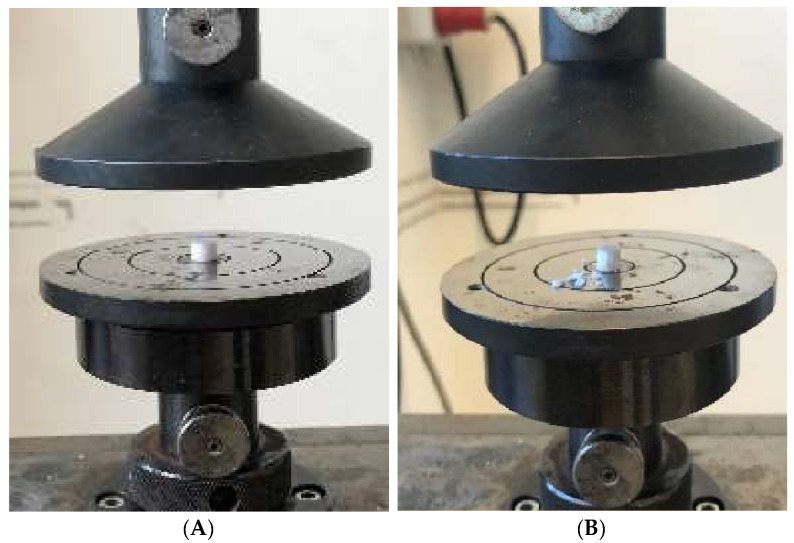
Test setup for the uniaxial compression test using the RMS implant as an example: (**A**) RMS implant with 6 mm height before the test; (**B**) RMS implant with 6 mm height after the test.

The unconfined compression test was performed on a Zwick ZMART.PRO universal testing machine (ZwickRoell GmbH & Co. KG, Ulm, Germany) with a 20 kN load cell according to DIN 51104:2010-08. Data were collected using Zwick’s testXpert II (version 3.7.1). The following parameters were used: Test speed 1 mm/min (position controlled), upper force limit of 8000 N, and maximum deformation of 50%. Three samples each were measured. The macroporous cylindrical β-TCP implant from curasan (Cerasorb M, curasan AG, Kleinostheim, Germany) and the microporous β-TCP implant (RMS) were each tested at a height of 6 mm and at the original height (curasan) of 20 mm. The microporous β-TCP implant (RMS) was shortened to 20 mm before the test in order to make it comparable with the Cursan implant. The samples were cut to the appropriate height using an EXAKT diamond band saw (EXAKT Advanced Technologies GmbH, Norderstedt, Germany). The hybrid structure was tested at its full height (9 mm). The test setup is shown in Figure 1.

#### 2.3.3. Degradation Tests

Three samples each were stored for 60 days in 5 mL Tris buffer with a pH value of 7.4 and an additional pH value of 5 was used to simulate the effects of an inflammatory reaction in the body at 37 °C in a Memmert drying oven (Memmert GmbH & Co. KG, Schwabach, Germany). The Tris buffer used was prepared according to the standard DIN EN ISO 10993-14 by dissolving 26.5 g of Tris in 1 L of double-distilled water, adjusting the pH to the desired values first with 10 mol/L HCl, then with 1 mol/L HCl and 1 mol/L NaOH, and then checking it with a WTW inoLab 7110 pH meter (Xylem Analytics Germany Sales GmbH & Co. KG, Weilheim, Germany) [28]. After incubation, all the scaffolds were rinsed with distilled water, dried, and weighed. The mechanical tests were then repeated as described in Section 2.3.2.

#### 2.3.4. Biocompatibility

MG-63 cells (ATCC, CRL 1427) were used for all biocompatibility testing. All the tests were performed with 50,000 cells/100 µL per scaffold. Per test, 10 identical scaffolds per geometry variation were used, and all the tests were repeated at least 3 times. All the scaffolds were cut to a height of 3 mm for biocompatibility testing.

*Live/Dead Assay*

On each scaffold and on Thermanox coverslips as a control, 100 µL of medium was pipetted with 50,000 MG-63 cells/100 µL. The well plates were then incubated for 2 h at 37 °C and 5% CO_2_ saturation in an incubator. After two hours, 1 mL of the specific cell medium described above was added to each well before the well plates were incubated in the incubator for 3, 7, and 10 days. The staining solution was prepared by adding 2 mL DPBS (Art. No. 14190-094, Gibco, Grand Island, NE, USA) to a Falcon tube (Greiner Bio-One International GmbH, Kremsmünster, Austria) and 4 µL ethidium homodimer III (Eth D-III) solution (together with the calcein part of the Live/Dead Cell Staining Kit II (PromoCell, Heidelberg, Germany)) according to the manufacturer’s protocol (PromoCell). After mixing the staining solution, 1 µL of calcein dye was added. All the steps were performed in the dark to avoid photobleaching of the staining solution and samples. To eliminate serum esterase activity, the medium was removed from all the samples at a given time point and the cells were washed. Staining was then performed according to a previously published protocol [27]. Evaluation was performed using an Olympus fluorescence microscope (BX51, Olympus, Osaka, Japan) at 5 different positions on the samples at 5× and 10× magnification.

*Cell Proliferation (WST-I)*

The cells were again seeded onto the scaffolds and, as a control, onto Thermanox coverslips in the same number and concentration as in the previous biocompatibility tests. After two hours of incubation in the incubator at 37 °C and 5% CO_2_ saturation, the cells adhered to the scaffolds and Thermanox coverslips (as a control) and the appropriate medium (1 mL) could be added. The plates were then incubated in the incubator for 3, 7, and 10 days. All of the medium was aspirated, and all the wells were washed three times with PBS. The scaffolds and Thermanox coverslips were then transferred to a new 24-well plate. In the old well plate, 300 µL of DMEM medium without phenol red (supplements: 1% FBS and 1% P/S) was added to each of the wells where the scaffolds and membranes were previously located. In the new well plate, 600 µL of the same medium was added to each of the wells containing the scaffolds and membranes. The blank contained only DMEM medium without phenol red (with the same additives) and was measured to account for background absorbance. Finally, 10% WST was added to all the samples at each time point (3, 7, 10 d) and incubated for 2 h. After 2 h, absorbance was measured using a spectrometer at λ = 450 nm.

*Cytotoxicity (LDH Assay)*

LDH measurements were performed at 24, 48, and 72 h. In addition to the scaffolds, positive controls (Triton X, 100% toxicity) and negative controls (cells only, 0% toxicity) were used for the measurements at different times. Both coated and uncoated scaffolds were used. The cells were seeded on the scaffolds and membranes in 100 µL of their medium (MG-63: 50,000 cells/100 µL). These were then incubated for 2 h in an incubator at 37 °C and 5% CO_2_ saturation. Subsequently, 1 mL of DMEM-F12 medium without phenol red was added to all the wells with the addition of 1% P/S and 1% FBS. As higher concentrations of FBS may induce background absorbance, only 1% FBS was used. In the positive controls (C+), an additional 1% Triton X-100 was added to kill 100% of the cells. After 24 h of incubation in the incubator, 100 µL from each well was transferred to 3 new wells of a 96-well plate. Thus, 3 wells of 100 µL each were obtained from 1 well. To ensure that the “blank” had the same concentration of phenol red, 100 µL of DMEM-F12 medium containing phenol red was added to this well prior to transfer. To assess cytotoxicity, the Cytotoxicity Detection Kit solution was prepared. For this, 111.1 µL of catalyst solution was mixed with 5 mL of staining solution. Of this, 100 µL was pipetted into each well before the well plate was incubated in the dark for 30 min. At the end of the 30 min period, the absorbance was measured at 490 nm using a spectrometer. The experiment was performed a total of 4 times.

### 2.4. Statistics

All the values in this paper are expressed as mean ± standard deviation. The calculations were performed using Origin 2023 Professional SR1 (OriginLab, Northampton, MA, USA). The Shapiro–Wilcox test was used to determine normal distribution, followed by ANOVA (Tukey test) for significance testing (*p* < 0.05).

## 3. Results

**Figure 2:**
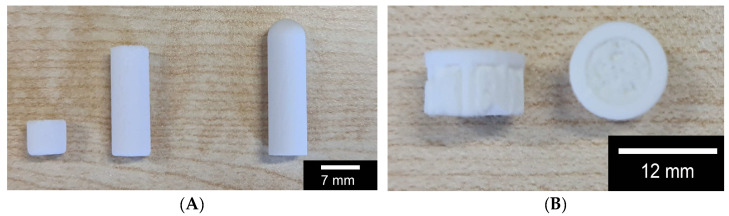
Overview of the scaffolds used: (**A**) macroporous β-TCP from Curasan (6 and 20 mm length) left and center; microporous β-TCP from RMS; (**B**) hybrid structure, side and top view; the RMS scaffold was shortened to 20 mm before the test in order to make it comparable with the Cursan scaffold.

For all further investigations, the different scaffolds (β-TCP RMS; β-TCP Curasan; hybrid structure) shown in Figure 2 below were used.

### 3.1. Porosity

The total porosity of the hybrid structure, including the differential porosity of the column structure and the freeze foam, was 74.4 ± 0.5%. The microporous β-TCP implant showed a porosity of 43.5 ± 2.4% in mercury porosimetry, while that of the macroporous β-TCP implant was 61.81%. An average pore radius of 2.69 µm was determined for the microporous β-TCP implant, with a scatter range between 2 and 5 µm. The average pore radius of the macroporous β-TCP implant was 18.5 µm, with a range of 3 to 80 µm (see Table 2).

### 3.2. µCT

**Figure 3:**
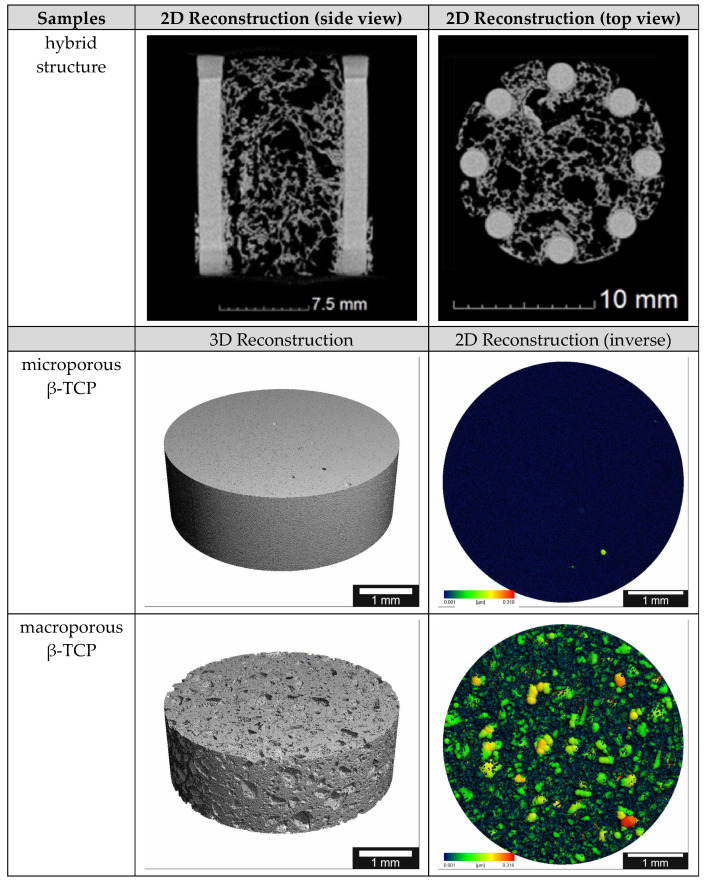
µCT reconstructions of the different scaffolds; the pore size ranges from 0.001 to 0.316 µm in the false color images.

Using µCT, the differences between the two TCP scaffolds (micro- vs. macroporous) can be easily shown. The following Figure 3 shows clear differences in the inverse 2D reconstruction. The microporous TCP scaffolds show a uniform pore structure, while the macroporous scaffolds have many pores of different sizes. The 3D reconstruction shows a similar picture (pls see Figure 3). In addition, the difference between the additive manufactured columns and the cold-foamed core of the hybrid structure is very easy to distinguish.

### 3.3. Mechanical Properties

**Figure 4:**
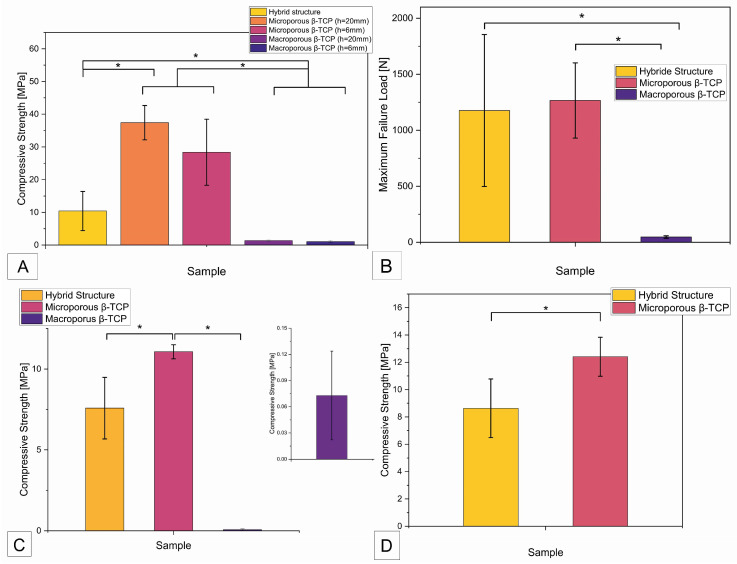
Overview of compressive strength and maximum failure load of the different samples. (**A**) Compressive strength as a function of sample origin; (**B**) maximum failure loads of the samples; (**C**) compressive strength of samples of different origin (manufacturing process) after degradation test according to ISO EN 10993-14 in Tris buffer with pH 7.4 for 60 days; (**D**) compressive strength of β-TCP samples (different production) after degradation in Tris buffer with pH 5.0 for 60 days; (*) statistically significant difference with *p* < 0.05.

When examining the compressive strength, there was a significant difference (*p* < 0.05) between the hybrid structure with a compressive strength of 10.4 ± 6 MPa and the two microporous β-TCP implants with a compressive strength of 37.4 ± 5.2 MPa (height 20 mm) and 28.4 ± 10.1 MPa (height 6 mm), as shown in Figure 4A below. Accordingly, there was no significant difference between the two tested heights of the microporous β-TCP implant. As expected, both macroporous β-TCP implants showed a very low compressive strength of 1.4 ± 0.2 MPa (height 20 mm) and 1.1 ± 0.3 MPa (height 6 mm), which was significantly different from all the other implants tested.

The maximum failure load refers to the force in N that the implant withstood before failure, regardless of the surface area and height of the implant. As shown in Figure 4B, the measurement of the maximum failure load showed a significant difference between the measured values of the hybrid structure of 1176.6 ± 678.7 N and the values of the macroporous β-TCP implant of 46.6 ± 11.1 N. The maximum failure load of the microporous β-TCP implant of 1266.4 ± 336.1 N was also significantly different from the measurement results of the macroporous implant. After degradation of the β-TCP implants in Tris buffer at pH 7.4 for 60 days, a significant difference (*p* < 0.05) was observed between the compressive strength of the hybrid structure at 7.6 ± 1.9 MPa and that of the microporous β-TCP implant at 11.1 ± 0.4 MPa (see Figure 4C). The compressive strength of the macroporous β-TCP implant was 0.07 ± 0.05 MPa. Compared to the initial values of the respective β-TCP samples, the compressive strength of the microporous β-TCP scaffold decreased significantly by 66% after the degradation test in Tris buffer. In contrast, the compressive strength of the hybrid structure and the macroporous β-TCP scaffold showed no significant change (see Table 3). Figure 4D shows the compressive strength of the scaffolds after degradation in Tris buffer at pH 5 for 60 days. The microporous β-TCP implant showed a significantly higher compressive strength of 12.4 ± 1.4 MPa compared to the compressive strength of the hybrid structure of 8.6 ± 2.1 MPa. All the results are summarized in Table 1 below.

### 3.4. Biocompatibility

#### 3.4.1. Live/Dead Assay

**Figure 5:**
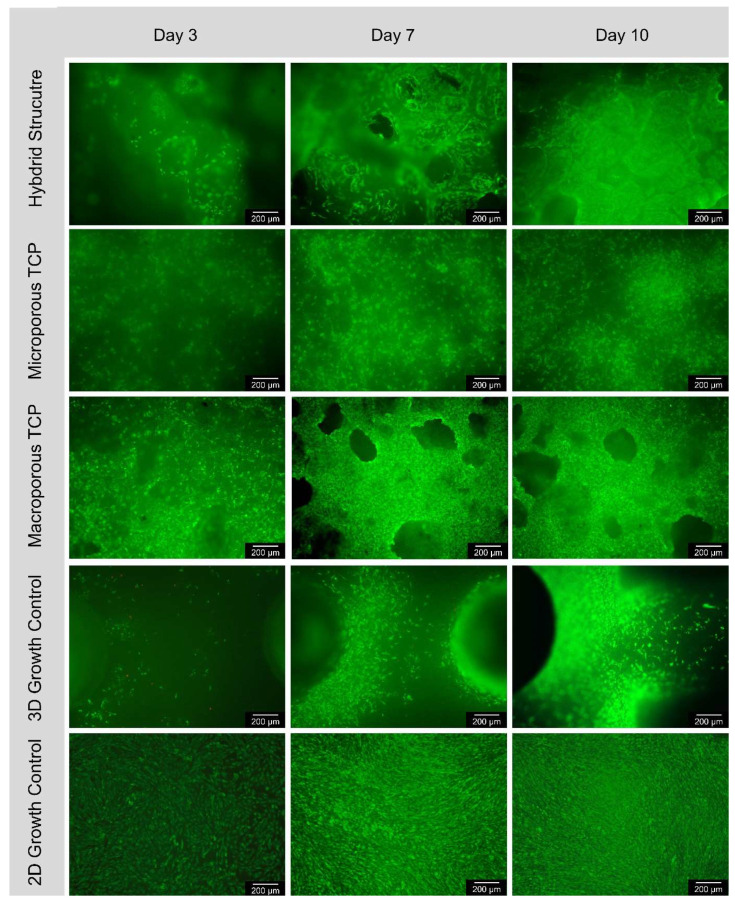
Course of cell growth on the surface according to 3, 7, and 10 days for hybrid structure; microporous TCP; macroporous TCP; 3D growth control and 2D growth control (Thermanox Coverslip); images in 5× magnification, images taken with Olympus BX-53 fluorescence microscope.

The following Figure 2 shows examples of fluorescence microscopy images of the surface of all the samples, magnified five times in the overlay filter. Accordingly, live cells fluoresce green and red cells fluoresce red. It is noticeable that the number of live cells increased over the 10-day experimental period. The number of cells per square millimeter on the surface of all the samples increased continuously over the 10-day test period. From day 3 to day 7, the average cell count of all the samples approximately doubled. As expected, the number of cells on the 2D control increased the most. The number of cells on the macroporous β-TCP scaffold had the second highest increase, but at significantly lower levels. The microporous β-TCP scaffold showed a steeper increase in cell number than the hybrid structure. The increase in cell number of the 3D control was intermediate. Only the 2D control showed a progressive increase in the number of dead cells per square millimeter on the surface of the specimens over the 10-day test period. In general, the number of dead cells was insignificant for all the samples (see Figure 5 and Table 4).

#### 3.4.2. Cell Proliferation (WST-I)

**Figure 6:**
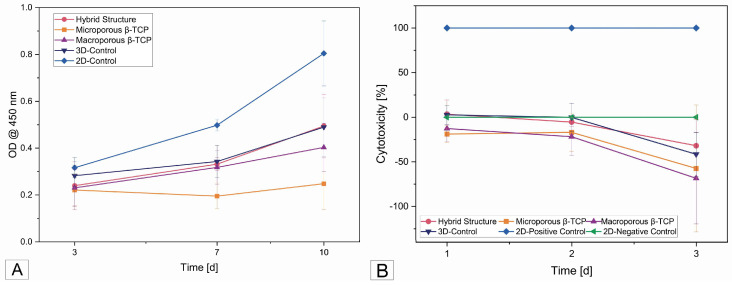
(**A**) Cell proliferation after 3, 7, and 10 days on the different samples; (**B**) cytotoxicity curves of all samples after 1, 2, and 3 days.

The absorbance values measured in the cell proliferation assay at a wavelength of 450 nm showed an increase between the first measurement time at 3 days and at 10 days for all the β-TCP implants and the 2D control (see Figure 6A). The graph of the microporous β-TCP implant showed the lowest slope, with no clear increasing trend at 3 days based on the absorbance measured at 7 days and the 3D control. As expected, the 2D control showed the strongest increase.

#### 3.4.3. Cytotoxicity (LDH)

The cytotoxicity of the scaffolds was 0% in almost all the samples. Only the 3D cell controls and the hybrid structure started with 2.6% and 3.5% cytotoxicity, respectively, on day 1 and then became zero at the next test time point. The negative values on the curve in Figure 6B correspond to 0% cytotoxicity.

## 4. Discussion

### 4.1. Porosity

Based on the present results, the influence of porosity on the mechanical strength of β-TCP implants could be demonstrated. This relationship is supported by the results of other authors who also found an inverse correlation between the porosity and compressive strength of β-TCP implants [29,30,31,32]. Peralta et al. [33] specifically addressed this relationship in human bone: a 5% increase in porosity corresponded to a 20 MPa decrease in compressive strength, which is approximately 10% of the maximum compressive strength of human cortical specimens. As mentioned above, the hybrid structure has a relatively high compressive strength for its high porosity of 74.4%; accordingly, the specific design of the column structure with macroporous filling based on the structure of human bone was successful in producing a predominantly microporous TCP scaffold with high mechanical resilience.

### 4.2. Mechanical Properties

The measurement of the maximum failure load of the macroporous β-TCP implant is in agreement with the previous investigations by Seidenstuecker et al. [28] and confirms the premise that the macroporous implant is only extremely limited for load-bearing applications. Compared to the compressive strength of human cancellous bone of 15.9 ± 7.4 MPa [34], the measured compressive strength of the macroporous scaffold of 1.2 ± 0.3 MPa is well outside the calculated standard deviation of the compressive strength of human bone.

In the first study to validate the tested microporous β-TCP implant, its compressive strength was investigated by Mayr et al. [25]. The maximum failure load of the implant was determined using a Zwick 1486 servo-hydraulic universal compression testing machine (ZwickRoell GmbH & Co. KG, Ulm, Germany) with a 20 kN load cell. The calculated compressive strength reflected the physiological conditions of the implant in the bone and was 79.19 ± 22.57 MPa. However, Mayr et al. [25] mechanically tested the bone-to-implant connection and not the implant alone.

In the case of the hybrid structure, the pressure resistance measurement of 10.4 ± 6 MPa in the present work differs from the results of Ahlhelm et al. [27] of 23 ± 4 MPa. This could be explained by the use of different measuring devices, the number of samples, and the environmental conditions. Ahlhelm et al. [27] performed their measurements using the Instron 8562 universal testing machine (Illinois Tool Works Inc., Norwood, USA). The results suggest that the hybrid structure of human cancellous bone is similar in terms of compressive strength. Furthermore, the increased compressive strength values of the microporous β-TCP implant of 32.9 ± 8.7 MPa are similar to those of Seidenstuecker et al. [28] of 24 ± 6 MPa. Accordingly, the compressive strength of the microporous β-TCP implant exceeds the compressive strength of human cancellous bone, which is estimated to be 15.9 ± 7.4 MPa [34]. In terms of compressive strength, the measured values of the hybrid structure most likely match the reference values of human cancellous bone and allow the assumption that this β-TCP implant can be used as an adequate replacement for cancellous bone in terms of mechanical strength. This is also suggested by the measured values of the microporous β-TCP implant, which are higher than those of human cancellous bone. However, human bone is known to consist of cancellous bone surrounded by cortical bone, which differs from cancellous bone in its mechanical properties. Therefore, the suitability of a bone implant must be tested or selected specifically for its intended use. For example, the requirements for the implant are different if it is to be implanted in an intact cortical scaffold, as a complete replacement of a tubular nose, or in a flat bone such as the pelvis.

The literature describes a decrease in the compressive strength of cortical bone as function of temperature and pressure on the pressure of the Havers channels. At a temperature of 38.5 °C, the compressive strength of the corticalis must decrease from 82.7 ± 11.3 MPa, measured along the course of the Havers channels, to 67.6 ± 10.9 MPa orthogonally [35]. The reported compressive strength of human cortex varies between 130 and 180 MPa [33,36]. As a result, the measured compressive strength values of all the β-TCP implants tested in the present study were found to be well below the reference value for the compressive strength of human cortical bone. Therefore, it can be assumed that the tested β-TCP implants would not be suitable for the expansion of a tube with loss of compactness at the defect site when operating with direct loads in the case of a critical bone substance defect. However, given the compressive strength of the hybrid structure, which is comparable to that of human cancellous bone, there is reason to believe that they could be used in cases of planned autologous cancellous bone grafting or when an inadequate autograft cannot be used. Mayr et al. [25] found in a large animal study that the microporous β-TCP implants with preoperatively applied autologous chondrocytes were resorbed at an average of 74% at 6 months and an average of 81% at 12 months.

The initially empty defect showed 15% bone content after 6 months, while the defect filled with the β-TCP implant was converted to 32% bone, as reported by Bernstein et al. [37] in the same study. From the beginning, the β-TCP implants were replaced by cancellous bone, which was built up according to Wolff’s law in the presence of stress. Based on these results, a more concrete statement can be made regarding the load-bearing capacity of the microporous β-TCP implant. The resorption and bone ingrowth behavior of the microporous β-TCP implant appears to be beneficial for the healing of osteochondral defects and to provide a better result in terms of the resulting bone quality and quantity than an unexpected empty defect. With regard to the hybrid structure, no concrete statements can be made regarding the load-bearing applicability of the implant, as the hybrid structure was used subdermally in the present in vivo study [27]. However, it can be assumed that the hybrid structure, due to its mechanical resilience, which is comparable to that of human cancellous bone, and its manufacture from pure beta-TCP, would exhibit similar resorption and bone incorporation behavior to that of the microporous beta-TCP implant and could therefore be used in a load-bearing capacity. In comparison with the literature, the values obtained for the compressive strength of the hybrid structure, taking into account the high porosity of the beta-TCP implant, are in the upper range of the comparison values. Compared to those of Santos et al. [38] at the same sintering temperature, the 3D printed β-TCP implants produced hybrid structure implants with a higher porosity of 74.4% compared to 54.4% and a higher compressive strength: 10.4 ± 6 MPa compared to 2.36 ± 0.05 MPa. When the sintering temperature was increased to 1400 °C and the porosity was 46.1%, Santos et al. [38] obtained comparable compressive strength values of 8.66 ± 0.11 MPa. Considering a slow cooling process, the increased sintering temperature only increased the crystallinity of β-TCP, but did not induce a phase transformation to α-TCP. The authors supported their statements with an X-ray diffraction analysis. Tarafder et al. [39] also reported similar values for their 3D printed microwave-sintered β-TCP implants with a compressive strength of 10.95 ± 1.28 MPa and a porosity of 42%. Liu et al. [32] compared a low compressive strength of 0.8 MPa to a maximum of 4.1 MPa with their 3D printed β-TCP implants by adjusting the porosity from 75% to 45% and the pore size from 1200 μm to 360 μm. The currently highest compressive strength values for 3D printed β-TCP implants were reported by Schmidleithner et al. [31]. The straight linear structure showed a compressive strength of 44.7 MPa at 50% porosity. When the porosity was increased to 75%, the reported value was reduced to 14.2 MPa. The compressive strengths of the hexagonal Kagome structure were 19.5 MPa and 6.75 MPa at 50% and 75% porosity, respectively. Schmidleithner et al. [31] also refer to the phenomenon of microcracks in β-TCP. Among other things, these microcracks could be responsible for the large scatter of these measured values, since they can lead to premature material fractures when printing is attempted [40]. Bertrand et al. [41] also reported this problem in relation to the wetting of their 3D printed β-TCP implants during the printing process. Using a 3D bioplotter, they produced cylindrical, multilayer β-TCP implants with a layer rotation of 1°, which were kept at a porosity of 38.8% between 14.97 ± 1.08 MPa and 41.6 ± 7.12 MPa. The maximum values were achieved with the 12-layer β-TCP implant printed with a 0.25 mm needle diameter after pretreatment in PBS. Compared to the compressive strength of the hybrid structure, the compressive strength of the microporous β-TCP implant was significantly higher. This was to be expected given the higher porosity of the hybrid structure, although the compressive strength of the implant is at the upper end of the literature range. It is clear that there are significant differences between the different manufacturing processes of β-TCP implants with respect to the mechanical strength of the implants. In the degradation test, there was no significant change between the compressive strength before and after the degradation test in Tris buffer at pH 7.4 for 60 days in the hybrid structure and the macroporous β-TCP implant. On the other hand, the compressive strength of the microporous β-TCP implant showed a significant reduction of 66% after the degradation test in Tris buffer at pH 7.4 for 60 days. Even after the degradation test in Tris buffer at pH 5, the measured value for the compressive strength of the hybrid structure did not differ significantly from the comparative value before the degradation test, while the compressive strength of the microporous β-TCP implant after degradation in Tris buffer at pH 5 for 60 days showed a significant decrease of 62% to 12.4 ± 1.4 MPa compared to the initial value before the degradation test. With regard to the macroporous β-TCP implant, it can be assumed that it would withstand a compressive load only under physiological conditions; there was no significant change in compressive strength after degradation in Tris buffer at pH 7.4 for 60 days compared to the initial value. After degradation in Tris buffer at pH 5 for 60 days, the macroporous β-TCP implant was degraded; so, it can be assumed that the implant would not remain as dimensionally stable in inflamed tissue. The absence of a significant reduction in the compressive strength of the hybrid structure after degradation in Tris buffer at pH 7.4 and pH 5 for 60 days supports the usability of this implant both in the physiological pH range of human blood and in inflamed tissue at acidic pH. The significant reduction in the compressive strength of the microporous beta-TCP implant after the degradation test in Tris buffer at pH 7.4 for 60 days, as well as in Tris buffer at pH 5, illustrates that the material properties of the implant change under physiological or inflammatory conditions and, in particular, that the compressive strength decreases. Due to the fact that the initial compressive strength value of the microporous beta-TCP implant is greater than the comparative compressive strength value for the compressive strength of human bone and that the values after degradation attempt to match the comparative value, the microporous beta-TCP implant is still considered for load-bearing applications under physiological or inflammatory conditions. It should be noted that the degradation test allowed a compression test after 60 days of incubation of the implants, whereas the cell culture only allowed a study up to a maximum duration of 10 days. This limitation of the cell culture leads to a noticeable deficit in data collection in the period of peri- and intra-implant bone regeneration, which for β-TCP extends over a total of more than 6 months [42]. In a direct comparison, it was shown that the compressive strength of the hybrid structure did not decrease under physiological and inflammatory conditions, whereas the compressive strength of the microporous β-TCP implant showed a significant decrease. The surprising finding of the lower pH sensitivity of the hybrid structure suggests that the foam of the hybrid structure, which has lower interconnectivity than the pores of the microporous β-TCP implant, represents a better sealed surface, which is why the acid can penetrate less.

### 4.3. Biocompatibility

Cell proliferation was detected on all β-TCP implants within 10 days using the cell proliferation assay and the live/dead assay. This observation is in agreement with the results of Ahlhelm et al. [27] and Mayr et al. [24], who characterized the hybrid structure and the microporous β-TCP implant as cell proliferation promoting and osteoconducting, respectively. In comparison, the microporous β-TCP implant showed a greater increase in cell number than the hybrid structure. One explanation for this may be that the cells migrated into the interior of the 3D construct due to the high porosity of the hybrid structure, meaning that a direct comparison of the increase in cell number over time in relation to the surface area of the construct may not be meaningful. It was found that the cells did not migrate into the microporous β-TCP implant, but only into the macroporous β-TCP implant and the hybrid structure. This was an expected result because the cells used do not fit into the micropores due to their size. There was also an increase in the number of cells located within the 3D constructs. Only a few cells were seen on the edge of the hybrid structure, i.e., on the microporous columnar structure.

This is consistent with the findings of Ghanaati et al. [43], who found that macropores combined with high overall implant porosity favored the ingrowth of cells and connective tissue fibers into the center of the β-TCP implant. Consequently, cell proliferation occurs on both the microporous β-TCP implant and the hybrid structure; however, the key difference is that numerous cells grow into the hybrid structure and proliferate within the construct. The hybrid structure proved to be extremely hygroscopic; i.e., during cell seeding, it was observed that the cell fluid was virtually sucked into the interior of the implant and that no droplets formed on the surface of the implant, as was the case with the other implants, except for the 3D control. This is a known phenomenon that Seidenstücker et al. [44] have already described with their β-TCP implants. This phenomenon is caused by the high total porosity of the hybrid structure of 74.4 ± 0.5% and the macropores of 0.1–0.6 mm diameter contained in the freezing foam.

As expected, the results of the lactate dehydrogenase assay with maximum cytotoxicity values of less than 20% at all time points for all the β-TCP implants indicate that the tested β-TCP implants are not cytotoxic. This is consistent with the data collected by Ahlhelm et al. [27] and Bernstein et al. [24]. In the context of the existing studies on the biocompatibility of β-TCP [27,45] and the present results, it can be assumed that the investigated β-TCP implants are not cytotoxic and allow cell proliferation on their surface. Therefore, the implants can be defined as osteoconductive.

Methodologically, the partially negative percentages for cytotoxicity can be explained by the experimental design. In the two-dimensional negative control, the drop of cell fluid was placed on the membrane and then incubated. On the 3D constructs, however, the drop of cell fluid could not be placed in such a way as to exclude the presence of cells at the bottom of the well. In order to make a statement about the cells on the 3D constructs only, they were transferred to a new well after the drop of cell fluid was placed. As a result, it is possible that there were initially fewer cells on the 3D constructs than on the negative control. The cells on the negative control may have proliferated to the point where there was insufficient space and medium for them to survive, resulting in more cells dying than on the 3D constructs. When calculating cytotoxicity, the value of the negative control is subtracted from the measured value of the sample; accordingly, the percentage value for cytotoxicity is negative if the measured value of the sample is less than the value of the negative control. As mentioned above, the negative values were considered as zero.

#### Limitations

In order to be comparable with previous measurements, we limited ourselves to unconfined compression measurements. Bending tests were not possible due to the limited size of the specimens (hybrid structure).

## 5. Conclusions

Both the conventionally fabricated microporous β-TCP scaffold and the 3D printed implant are suitable for bone replacement that is primarily stable during motion and loading. In addition, both β-TCP scaffolds were found to be biocompatible and osteoconductive, as expected from their manufacture from β-TCP. In order to assess the potential of the two β-TCP scaffolds for bone replacement in load-bearing applications, further studies over a longer period of time are required to observe the resorption and bone ingrowth behavior of the implants, including histological examination, to ensure the mechanical load-bearing capacity of the implants during the bone remodeling process. The shear strength of the two implants could also be studied in more detail.

## 6. Outlook

Advances in additive manufacturing clearly indicate that the production of scaffolds and patient-specific implants will clearly shift to this area. It will then be necessary to test these new scaffolds/implants for biocompatibility and mechanical properties. In particular, the mechanical properties must be matched to those of the bone to avoid problems such as stress shielding.

## Figures and Tables

**Table 1:** Sample characteristics and dimensions.

	Characteristics	Dimensions [mm]
Microporous TCP short	Pore size between 1 and 10 µm	Ø 7 × 6
Microporous TCP long		Ø 7 × 20
Macroporous TCP short	Pore size larger than 100 µm	Ø 7 × 6
Macroporous TCP long		Ø 7 × 20
Hybrid structure	Additive manufactured capitals and columns, cold-foamed core	Ø 12 × 9

**Table 2:** Overview of porosity and pore radius for the different samples.

Sample	Porosity [%]	Pore Radius [µm]
hybrid structure	74.4 ± 0.5	-
microporous β-TCP	43.5 ± 2.4	2.69
macroporous β-TCP	61.8 ± 0.9	18.5

**Table 3:** Summary of the compressive strengths and maximum failure loads of the various β-TCP scaffolds produced (with/without incubation in TRIS).

Compressive Strength [MPa]			
Sample	Hybrid Structure	Microporous β-TCP	Macroporous β-TCP
No Tris buffer	10.4 ± 6	32.9 ± 8.7	1.2 ± 0.3
Tris buffer pH 7.4	7.6 ± 1.9	11.1 ± 0.4	0.07 ± 0.05
Tris buffer pH 5	8.6 ± 2.1	12.4 ± 1.4	n.a.
**Maximum Failure Load [N]**			
**Sample**	**Hybrid Structure**	**Microporous β-TCP**	**Macroporous β-TCP**
No Tris buffer	1176.6 ± 678.7	1266.4 ± 336.1	46.6 ± 11.1
Tris buffer pH 7.4	856.9 ± 215.4	425.7 ± 16.7	2.8 ± 2
Tris buffer pH 5	975.7 ± 243.1	477.5 ± 55	n.a.

**Table 4:** Cells per mm^2^ on the different scaffolds.

Surface	Day 3		Day 7		Day 10	
Cells/mm^2^	Living	Dead	Living	Dead	Living	Dead
Hybrid structure	66 ± 22	2 ± 4	131 ± 66	3 ± 5	240 ± 84	1 ± 1
Microporous β-TCP	128 ± 136	1 ± 1	266 ± 270	1 ± 1	624 ± 462	1 ± 3
Macroporous β-TCP	256 ± 299	1 ± 1	520 ± 520	0	993 ± 748	3 ± 9
3D control curasan	64 ± 70	9 ± 11	166 ± 101	12 ± 22	380 ± 216	6 ± 4
2D control Thermanox	862 ± 548	3 ± 4	1697 ± 403	12 ± 15	2468 ± 420	50 ± 51

## Data Availability

The data presented in this study are available on request from the corresponding author.
